# Identification and characterization of genome-wide resistance gene analogs (RGAs) of durian (*Durio zibethinus* L.)

**DOI:** 10.1186/s43141-022-00313-8

**Published:** 2022-02-14

**Authors:** Cris Q. Cortaga, Romnick A. Latina, Rosteo R. Habunal, Darlon V. Lantican

**Affiliations:** 1 Institute of Plant Breeding (IPB), College of Agriculture, University of the Philippines Los Baños, 4031 College, Laguna, Philippines; 2 Institute of Weed Science, Entomology, and Plant Pathology (IWEP), College of Agriculture and Food Science, University of the Philippines Los Baños, 4031 College, Laguna, Philippines

**Keywords:** Durian, Genome-wide, Resistance gene analogs (RGAs), Plant defense, Bioinformatics

## Abstract

**Background:**

Durian (*Durio zibethinus* L.) is a tropical fruit crop which is popular in Southeast Asia but recently gaining popularity in other parts of the world. In this study, we analyzed the resistance gene analogs (RGAs) of durian through mining of the currently available reference genome of its ‘Musang King’ cultivar (PRJNA400310).

**Results:**

A total of 2586 RGAs were identified in the durian genome consisting of 47 nucleotide binding site proteins (NBS), 158 NBS-leucine rich repeat proteins (NL), 400 coiled-coil NBS-LRR (CNL), 72 toll/interleukin-1 receptor NBS-LRR (TNL), 54 coiled-coil NBS (CN), 10 toll/interleukin-1 receptor NBS (TN), 19 toll/interleukin-1 receptor with unknown domain (TX), 246 receptor-like proteins (RLP), 1,377 receptor-like kinases (RLK), 185 TM-CC, and 18 other NBS-containing proteins with other domains. These RGAs were functionally annotated and characterized via gene ontology (GO) analysis. Among the RGAs with the highest copies in durian genome include the putative disease resistance RPP13-like protein 1, disease resistance protein At4g27190, disease resistance protein RPS6, Probable disease resistance protein At4g27220, and putative disease resistance protein RGA3, while 35 RGAs were found to be novel. Phylogenetic analyses revealed that the genome-wide RGAs were broadly clustered into four major clades based on their domain classification.

**Conclusion:**

To our knowledge, this is the most comprehensive analysis of durian RGAs which provides a valuable resource for genetic, agronomic, and other biological research of this important tropical fruit crop.

**Supplementary Information:**

The online version contains supplementary material available at 10.1186/s43141-022-00313-8.

## Introduction

Plants sense insect pests and pathogen invasion via pathogen recognition receptors (PRRs) in the cell, whereas attacker-specific effectors are identified via a gene-for-gene interaction through resistance (R) proteins [1–3]. The PRRs and R genes are referred to as resistance gene analogs (RGAs) which share conserved domains and motifs [4]. They are in charge of intracellular signaling and turning on plant defense genes. PRRs are made up of membrane-associated RLKs and RLPs. RLKs have an extracellular sensing domain, such as a leucine-rich repeat (LRR) domain or a lysin motif (LysM) domain, a transmembrane (TM) domain, and an intracellular kinase domain, whereas RLPs have a similar structure except for the absence of an intracellular kinase domain [5]. The R proteins are intracellular effector-recognition receptors and contain certain domains/motifs such as serine/threonine kinases, nucleotide binding sites (NBS), LRRs, TMs, leucine-zipper, coiled-coil (CC), and toll/interleukin-1 receptor (TIR) [4, 6, 7]. Among these, majority of R proteins belong to NBS-LRR class. Meanwhile, the subgroups of NBS-encoding proteins are designated as NBS, CNL, TNL, CN, TN, NL, TX, and other NBS protein that shows chimeric domain/motif architecture.

The advent of genomics technologies facilitated the development of DNA markers tagging economic traits, characterization of diverse protein families, and discovery of novel biological insights into numerous species at the genome-wide scale [8–11]. As a useful tool for resistance breeding, the RGAs have been widely studied to obtain a deeper insight on the underlying molecular defenses of the plant. Since RGAs in plants have conserved structural properties, bioinformatics investigations of next-generation sequencing (NGS) data may be used to undertake comprehensive RGA prediction [12–15]. Several studies have exemplified the utility of these RGAs as a rich source of functional markers not just for tagging pest resistant loci for many crops [16] for plant molecular breeding [17] but also for genetic structure and diversity analyses [18].

Durian (*Durio zibethinus* L.) is a tropical fruit crop grown in Southeast Asia known for its distinct taste and aroma. Also hailed as the “king of fruits”, it has started to gain popularity in the USA and other parts of the world leading to an increasing economic market value. Several studies have also proven its high nutritional and nutraceutical potential [19]. However, the primary restrictions to obtaining optimal durian production include diseases such as root rot, stem rot, and fruit rot, as well as insect pests [20]. Recently, the whole genome of durian (c.v. Musang King) with a haploid size of 738 Mb had been published [21]. The availability of its genomic reference has paved the way to more in-depth research opportunities for durian, such as those related to understanding insect and pathogen resistance. Through mining of the currently released durian genome, the genome-wide RGAs of durian were identified and characterized in this paper. To the best of our knowledge, this work covers the most comprehensive identification, characterization, and evolutionary investigation of durian RGAs.

## Materials and methods

### Identification and classification of durian RGAs

The predicted gene models from the whole genome of durian were accessed from Teh et al. (2017) (NCBI BioProject PRJNA400310) for RGA analysis. Using the automated RGA prediction pipeline RGAugury [4], the genome-wide RGA of durian belonging to membrane associated RLK and RLP families, and NBS and TM-CC containing proteins were identified in the gene models from the annotated durian genome. Using an *e*-value cut-off of 1e–5, the input protein sequences were filtered using a BLASTp search against the RGAdb database of the RGAugury software package.

### Characterization and annotation of durian RGAs

The RGAs of durian were functionally annotated using the BLAST2GO package [22]. The homology of the protein sequences of each predicted RGAs was determined through BLASTp analysis (with *e*-value of 1e–5) using the UniProtKB/SwissProt protein database. The mapped BLAST hits were then merged to InterProScan [23] search output to produce the gene ontology (GO) annotations, such as the molecular function (MF), biological processes (BP), and cellular component (CC), which were designated to each RGA protein identified from the whole genome of durian.

### Evolutionary analysis of durian RGAs

Multiple sequence alignment was done using the FASTA amino acid sequences of the genome-wide RGAs of durian as input using the CLUSTALW program [24] with the following parameters: Gap Opening Penalty: 10; Gap Extension Penalty: 0.2. The maximum likelihood statistical approach in IQ-TREE [25] was used to construct phylogenetic of the aligned protein sequences, with the best-fit substitution model selected using ModelFinder [26] according to the Bayesian information criterion (BIC). The phylogenetic tree was generated with 1000 iterations of ultrafast bootstrapping [27] using a generic matrix (JTT) with empirical amino acid frequencies (+F) and discrete Gamma (+G4) rate heterogeneity across sites. FigTree (v1.4.4) [28] was used to display and preprocess the phylogenetic tree that had been constructed.

## Results and discussion

### Identification of RGAs

RGAugury [4], an efficient integrative bioinformatics pipeline for predicting RGAs in plants using NGS data, was used to identify RGAs from the retrieved gene models of the whole genome of Musa King durian variety [21]. Durian RGAs are made up of 47 NBS, 400 CNL, 72 TNL, 54 CN, 10 TN, 158 NL, 19 TX, 246 RLP, 1377 RLK, 185 TM-CC, and 18 other NBS-containing proteins with other domains, totaling 2586 RGAs (Table 1; Additional file 1). When the genome-wide RGAs of durian were compared to the predicted RGAs reported from other plant species [13], it was revealed that it has the most RGAs (2586), followed by peach (2005), orange (1806), mango (1775), rice (1537), cacao (1171), Arabidopsis (979), corn (935), tomato (922), banana (769), and finally, papaya (402). (Table 1). The RGA content and characteristics of a plant have been linked to resistance [29], and the high number of RGAs in durian may imply substantial innate plant resistance.

**Table 1:** Distribution and of genome-wide RGAs of durian compared to other sequenced plant genomes

RGA codes	Durian (***Durio zibethinus***)^**a**^	Mango (***Mangifera indica***)^**b**^	Peach (***Prunus persica***)^**b**^	Papaya (***Carica papaya***)^**b**^	Cacao (***Theobroma cacao***)^**b**^	Orange (***Citrus sinensis***)^**b**^	Arabidopsis (***Arabidopsis thaliana***)^**b**^	Tomato (***Solanum lycopersicum***)^**b**^	Rice (***Oryza sativa***)^**b**^	Banana (***Musa acuminata***)^**b**^	Corn (***Zea mays***)^**b**^
NBS	47	54	45	10	18	60	9	58	45	8	16
CC-NBS-LRR (CNL)	400	242	185	10	187	158	48	74	223	50	69
TIR-NBS-LRR (TNL)	72	79	157	4	15	31	98	23	0	0	0
CC-NBS (CN)	54	78	5	5	19	65	1	5	37	7	12
TIR-NBS (TN)	10	30	19	1	3	31	16	8	3	2	3
NBS-LRR (NL)	158	107	183	22	67	189	29	93	197	43	52
TIR with unknown domain (TX)	19	45	44	5	7	49	40	9	3	3	3
Other NBS-encoding	18	7	30	3	0	7	17	1	0	0	0
RLP	246	133	176	25	159	215	65	77	102	95	35
RLK	1377	917	1040	282	634	895	591	507	856	489	649
TM-CC	185	83	121	35	62	106	65	67	71	72	96
**Total RGAs**	2586	1775	2005	402	1171	1806	979	922	1537	769	935

^a^Inferred from this paper

^b^From Lantican et al. (2020) [13]

**Fig. 1:**
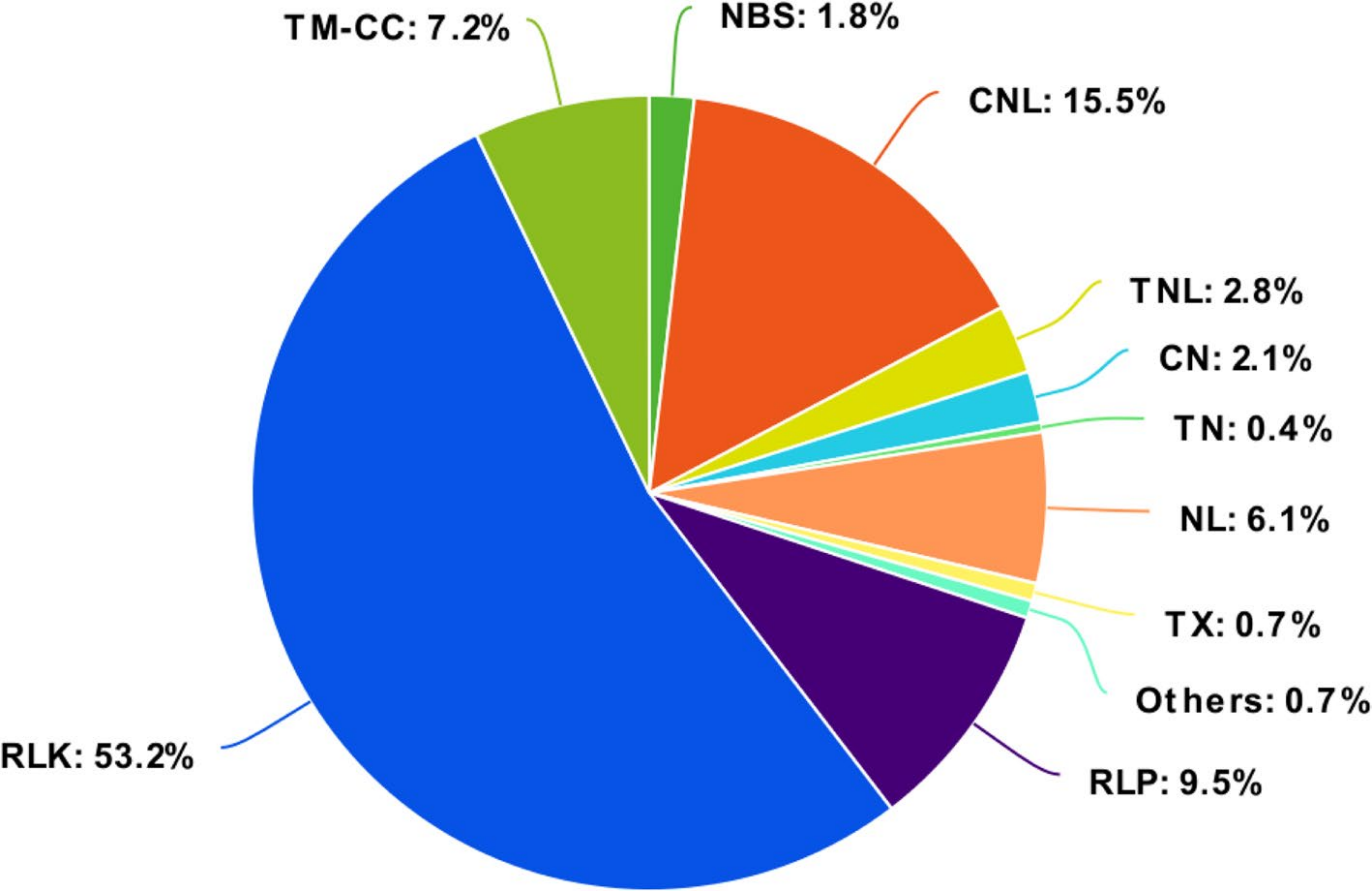
Percentage and distribution of the classifications based on conserved domains and motifs of genome-wide RGAs of durian

Among the RGAs investigated, the RLK was the most common group comprising the majority of RGAs found in the analyzed plant genomes (Table 1). In durian, 1377 RLK genes were identified which accounted for half (or 53.2%) of the predicted RGAs followed by 778 NBS-encoding genes (30.1%) (Table 1, Fig. 1). The NBS-encoding genes are the frequent targets for *R* gene analysis and, thus, are the best-known and well-identified family of RGAs. In the pineapple genome, 177 NBS-encoding genes were identified [14] while 352 NBS-encoding genes were identified in the sunflower genome [7]. As a dicot species, durian contains all NBS-encoding proteins (NBS, CNL, TNL, CN, TN, NL, TX, and other NBS-encoding proteins), unlike monocots (e.g., rice, banana, and corn), which typically lack the TNL protein (Table 1) [30]. TNL genes are thought to have been lost from the monocot lineage following the divergence of dicots and monocots [14]. Eighteen putative RGAs were designated as “other” NBS-encoding proteins (Table 1) because they had chimeric domain/ motif architecture, i.e., unexpected domain combination of TIR and CC domains [4]. In terms of RLPs and TM-CC, the durian genome contains 246 and 185 genes, respectively or 9.5% and 7.2% of the total durian RGAs, respectively (Table 1, Fig. 1).

### GO functional annotation of RGAs

**Fig. 2:**
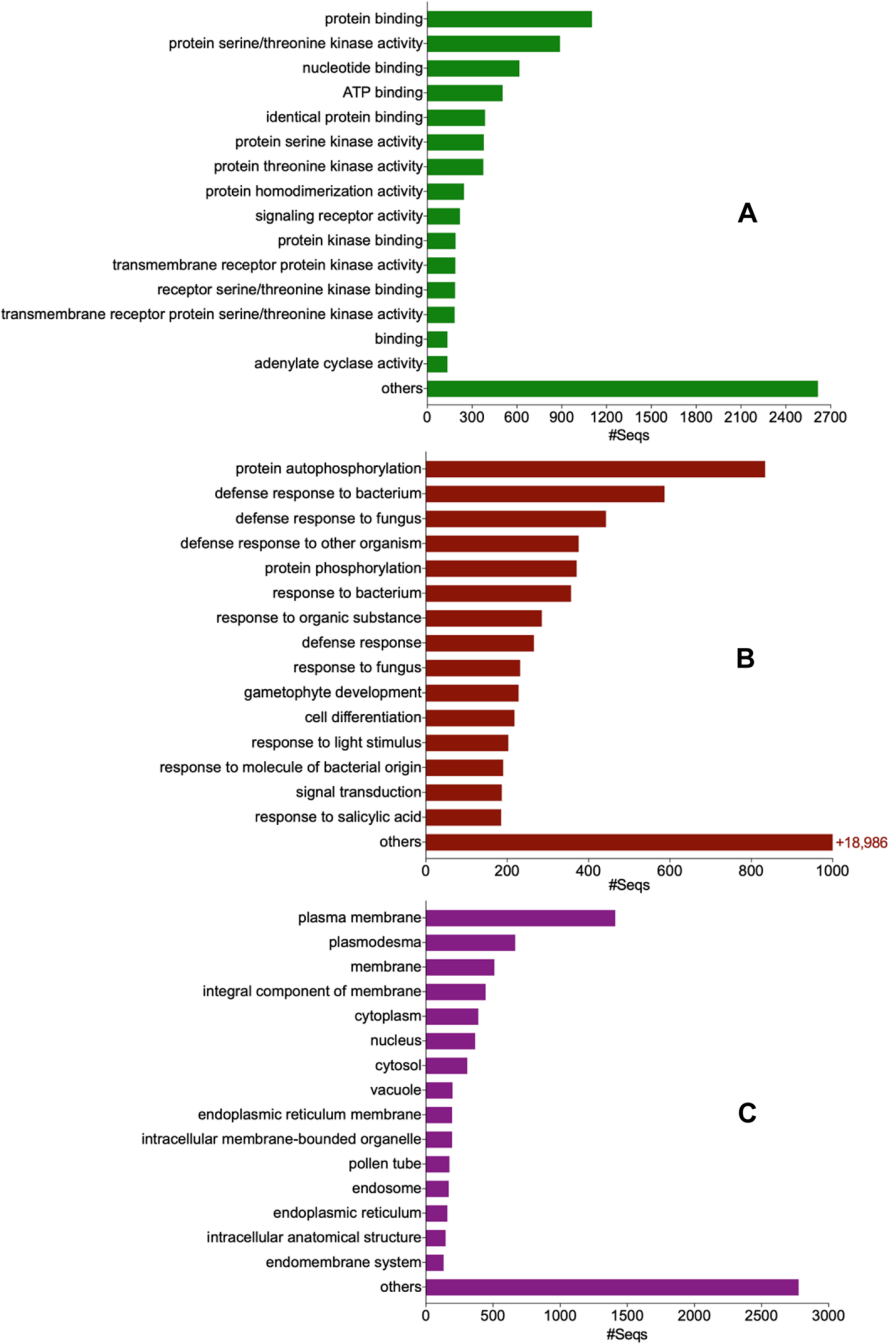
Distribution of GO annotation of genome-wide RGAs of durian based on molecular function (**a**), biological processes (**b**), and cellular component (**c**)

GO analysis was used to determine the associated molecular functions, biological processes, and cellular localizations of the durian genome-wide RGAs. As most RGAs are extra- and intracellular binding receptors that modulate cellular defense signaling via a cascade of kinase activities [31], their molecular functions are primarily associated with protein/nucleotide binding and kinase activity (Fig. 2a; Additional file 2a). On the other hand, the biological processes of RGAs are extremely diverse, resulting in a wider array of GO terms. The RGAs are primarily involved in protein autophosphorylation during cellular signal transductions, defense/resistance/immune responses to various stresses caused by biotic (e.g., insects and diseases) and abiotic (e.g., water deprivation, salt stress, UV stress) factors, and in various plant growth and development processes (from embryonic to floral/pollen development) (Fig. 2b; Additional file 2b).

Several RGAs are also involved in hormone-mediated signaling pathways and systemic acquired resistance which include the phytohormones abscisic acid, jasmonic acid, auxin, salicylic acid, gibberellic acid, ethylene, cytokinin, and brassinosteroids (Additional file 2b). Crosstalk between these plant hormones is critical for modulating defense signaling and activating systemic resistance against pathogens and insect pests [32]. As expected, RGAs are predominantly located in the cell's membranes, plasmodesma, cytoplasm/cytosol, and nucleus (Fig. 2c; Additional file 2c), as these are important recognition sites for pathogen/insect attack and effector proteins. The RGAs in these cellular components operate to transform extracellular stimuli into intracellular responses for defense activation.

### Durian resistance (R)/defense proteins

The durian genome-wide RGAs exhibited similarity to a wide range of well-known R/defense proteins (Table 2; Additional file 3). Among these predicted RGA proteins are LRK10L/Lr10 resistance proteins against leaf rust caused by *Puccinia triticina* [33]; RPP resistance proteins against downy mildew caused by *Peronospora parasitica* [34]; Resistance proteins R1-A and RGA/RGA-blb protect against the catastrophic late blight disease brought by *Phytophthora infestans* [35]; RRS1 resistance proteins (probable WRKY transcription factor) against *Colletotrichum higginsianum* and *Ralstonia solanacearum* [36]; ToMV resistance protein Tm-2(2) against certain tobamoviruses including, tomato mosaic virus (ToMV) and tobacco mosaic virus (TMV) [37]; RPS and RPM1 resistance proteins against the biotrophic pathogen *Pseudomonas syringae* [38]; and ERECTA protein for quantitative resistance to *Ralstonia solanacearum* bacterial wilt and the necrotrophic fungus *Plectosphaerella* [39]. In plants, this protein is also involved in the regulation of efficient transpiration [40].

**Table 2:** Durian RGAs with homology to well-known resistance proteins

Recommended name	Alternative name	Domain	No. of copies
Putative disease resistance RPP13-like protein 1		CN, CNL, NBS, NL	207
Disease resistance protein At4g27190		CN, CNL, NBS, NL, OTHER	135
Disease resistance protein RPS6	Resistance to Pseudomonas syringae 6	NL, OTHER, TN, TNL, TX	94
Probable disease resistance protein At4g27220		CNL, NBS, NL, OTHER	66
Putative disease resistance protein RGA3	Blight resistance protein B149; RGA1-blb	CN, CNL, NBS, NL, RLP	53
Putative disease resistance protein RGA1	RGA3-blb	CN, CNL, NBS, NL	37
Disease resistance protein RPM1	Resistance to Pseudomonas syringae protein 3	CNL, NL	16
Probable disease resistance protein At1g15890		CN, NBS	15
Disease resistance protein RPS4	Resistance to Pseudomonas syringae 4	CNL, NL, TNL, RLP	13
Putative disease resistance protein RGA4	RGA4-blb	CN, CNL, NBS, NL	13
Disease resistance protein SUMM2	Disease resistance protein At1g12280; Protein SUPPRESSOR OF MKK1 MKK2 2	CN, CNL	12
Probable disease resistance protein At5g63020	pNd11	CN, CNL	12
Disease resistance protein RGA2	Blight resistance protein RPI; RGA2-blb	CN, NL	9
Disease resistance protein RPS5	Resistance to Pseudomonas syringae protein 5; pNd3/pNd10	CN, CNL, NL	9
Putative disease resistance protein At1g50180		CNL, NL	9
Probable disease resistance protein At1g61190		CN, NBS	7
LEAF RUST 10 DISEASE-RESISTANCE LOCUS RECEPTOR-LIKE PROTEIN KINASE-like 1.2	Probable receptor-like serine/threonine-protein kinase LRK10L-1.2	RLK	6
Disease resistance protein RRS1	Disease resistance protein RCH2; Probable WRKY transcription factor 52; Resistance to Colletotrichum higginsianum 2 protein; Resistance to Ralstonia solanacearum 1 protein	NBS, TN	5
LEAF RUST 10 DISEASE-RESISTANCE LOCUS RECEPTOR-LIKE PROTEIN KINASE-like 1.3	Probable receptor-like serine/threonine-protein kinase LRK10L-1.3	RLK	5
Probable disease resistance protein At5g66900		CNL, NL	5
Putative disease resistance protein At4g10780		CN, CNL, NL	5
Rust resistance kinase Lr10	Probable receptor-like serine/threonine-protein kinase LRK10	RLK	5
Disease resistance RPP13-like protein 4	Disease resistance protein ZAR1; Protein HOPZ-ACTIVATED RESISTANCE 1; AtZAR1	CNL, NL	4
LEAF RUST 10 DISEASE-RESISTANCE LOCUS RECEPTOR-LIKE PROTEIN KINASE-like 2.1	Probable receptor-like serine/threonine-protein kinase LRK10L-2.1	RLK	4
Putative disease resistance protein At3g14460		CNL	4
Disease resistance protein RPS2	Resistance to Pseudomonas syringae protein 2	CNL, NBS	3
Probable disease resistance protein At1g58390		CNL, NL	3
Probable disease resistance protein At1g61300		CN, NBS	3
Putative disease resistance protein At1g63350		CN, NL	3
Disease resistance protein RFL1	RPS5-like protein 1; pNd13/pNd14	CN	2
Disease resistance protein RPP8	Resistance to Peronospora parasitica protein 8	NL	2
Disease resistance RPP8-like protein 3		NL	2
LEAF RUST 10 DISEASE-RESISTANCE LOCUS RECEPTOR-LIKE PROTEIN KINASE-like 1.5	Probable receptor-like serine/threonine-protein kinase LRK10L-1.5	RLK	2
LRR receptor-like serine/threonine-protein kinase ERECTA	Protein QUANTITATIVE RESISTANCE TO PLECTOSPHAERELLA 1; Protein QUANTITATIVE RESISTANCE TO RALSTONIA SOLANACEARUM 1; Protein TRANSPIRATION EFFICIENCY 1	RLK	2
Probable disease resistance protein At4g33300		NL	2
Probable disease resistance RPP8-like protein 4		NL	2
Disease resistance protein RRS1	Disease resistance protein RCH2; Disease resistance protein SLH1; Probable WRKY transcription factor 52; Protein RPS4-homolog; Protein SENSITIVE TO LOW HUMIDITY 1; Resistance to Colletotrichum higginsianum 2 protein; Resistance to Ralstonia solanacearum 1 protein; WRKY DNA-binding protein 52	NBS	1
Disease resistance protein UNI	Protein UNI	CN	1
Late blight resistance protein R1-A; Protein R1		NBS	1
LEAF RUST 10 DISEASE-RESISTANCE LOCUS RECEPTOR-LIKE PROTEIN KINASE-like 1.1	Probable receptor-like serine/threonine-protein kinase LRK10L-1.1	RLK	1
LEAF RUST 10 DISEASE-RESISTANCE LOCUS RECEPTOR-LIKE PROTEIN KINASE-like 2.5	Probable receptor-like serine/threonine-protein kinase LRK10L-2.5	RLK	1
LEAF RUST 10 DISEASE-RESISTANCE LOCUS RECEPTOR-LIKE PROTEIN KINASE-like 2.8	Probable receptor-like serine/threonine-protein kinase LRK10L-2.8	RLK	1
Probable disease resistance protein At1g52660		NBS	1
Probable disease resistance protein At5g45440		NBS	1
Probable disease resistance protein At5g47260		NBS	1
Probable disease resistance RPP8-like protein 2		NL	1
ToMV resistance protein Tm-2(2)	Disease resistance protein Tm-2(2); ToMV resistance protein Tm-2a	CNL	1

The durian RGAs also shared homology with the SUMM2 protein (SUPPRESSOR OF mkk1 mkk2 2), which is triggered when the pathogen effector HopAI1 disrupts the MEKK1-MKK1/MKK2-MPK4 cascade in the basal defense response [41, 42]. The disease resistance protein UNI, which is implicated in disease resistance by exhibiting constitutive expression of pathogenesis-related genes via the salicylic acid (SA) signaling pathway, was also found [43, 44]. It is also vital to the development of shoot architecture via the cytokinin signaling system [43, 44]. Homology to the Disease resistance protein RFL1, a RPS5-like protein 1, was also identified in durian RGA (Table 2). Other R proteins present in durian revealed similarity to a number of putative disease resistance proteins from *Arabidopsis thaliana* (At) that have yet to be extensively investigated (Table 2).

Among these well-known resistance proteins, the five highest copies in the durian genome include the putative disease resistance RPP13-like protein 1 (207 copies), disease resistance protein At4g27190 (135 copies), disease resistance protein RPS6 (94 copies), Probable disease resistance protein At4g27220 (66 copies), and putative disease resistance protein RGA3 (53 copies) (Table 2). Further filtering of genome-wide RGAs linked with the GO term “insect response” (GO:0009625) revealed 15 RGAs that may play key roles in insect defense pathways (Table 3). Notably, all of these RGAs were shown to have RLK domains, as was also observed in mango [13], where insect responsive RGAs possess RLK/RLP domains. On the other hand, 35 RGAs were revealed to be novel, or their biological functions have not yet been investigated in durian (Additional file 3).

**Table 3:** Durian RGAs with homology to proteins in UniProtKB/SwissProt database with “response to insect” GO annotation

Recommended name	Alternative name	Domain	No. of copies
Probable LRR receptor-like serine/threonine-protein kinase At1g56130		RLK	15
Probable LRR receptor-like serine/threonine-protein kinase At1g07650		RLK	15
Probable leucine-rich repeat receptor-like serine/threonine-protein kinase At3g14840		RLK	13
Probable LRR receptor-like serine/threonine-protein kinase RFK1	Receptor-like kinase in flowers 1	RLK	10
Probable LRR receptor-like serine/threonine-protein kinase At1g53430		RLK	2
Probable LRR receptor-like serine/threonine-protein kinase At1g53440		RLK	2
Probable serine/threonine-protein kinase At1g01540		RLK	2
Probable LRR receptor-like serine/threonine-protein kinase RKF3	Receptor-like kinase in flowers 3	RLK	2
C-type lectin receptor-like tyrosine-protein kinase At1g52310		RLK	2
Cysteine-rich receptor-like protein kinase 3; Cysteine-rich RLK3		RLK	1
Calmodulin-binding receptor kinase CaMRLK;	Calmodulin-binding receptor-like kinase; AtCaMRLK; Protein MATERNAL EFFECT EMBRYO ARREST 62	RLK	1
Probable LRR receptor-like serine/threonine-protein kinase At1g56140		RLK	1
MDIS1-interacting receptor like kinase 2; AtMIK2	Probable LRR receptor-like serine/threonine-protein kinase At4g08850	RLK	1
LysM domain receptor-like kinase 4; LysM-containing receptor-like kinase 4		RLK	1
Probable receptor-like protein kinase At1g11050		RLK	1

### Evolutionary relationships of RGAs

**Fig. 3:**
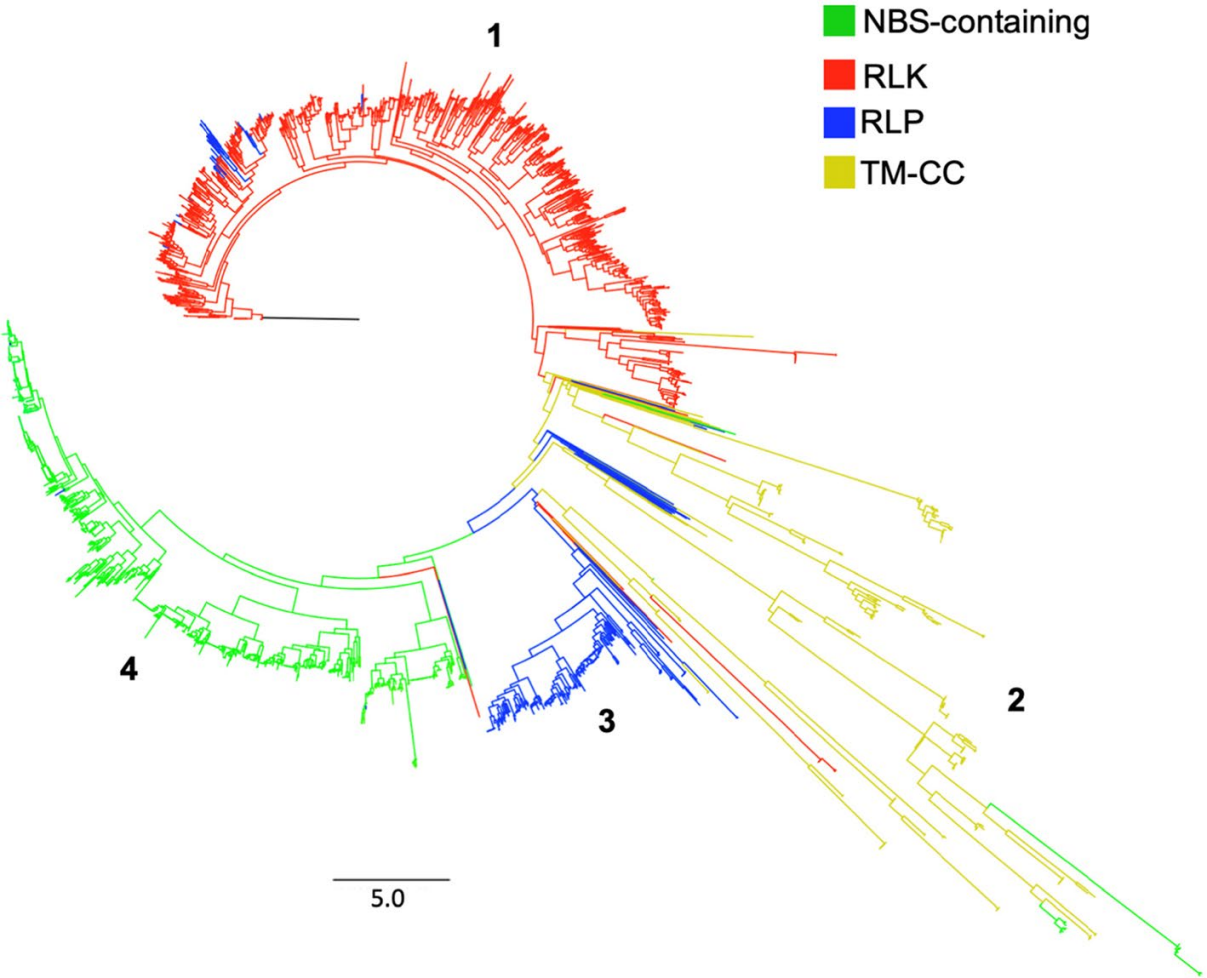
Maximum likelihood phylogenetic tree constructed from the sequence alignment of genome-wide RGAs of durian

To investigate the evolutionary relationships and diversity of durian RGAs, a maximum likelihood phylogenetic tree (Fig. 3) was built using the best-fit model determined based on BIC (Additional file 4). The RGAs were mostly clustered based on their conserved domains and motifs, indicating four major clades (corresponding to four major RGA families) with subclades from other RGA domains (Fig 3). Clade 1 is mostly made up of RLKs, with subclades that include RLPs and TM-CC. Clade 2 is dominated by TM-CC, with several subclades from all other RGA domains. Unlike in the TM-CC proteins derived from genomic sequences which form a distinct clade, the TM-CC proteins derived from transcriptomic sequences can form widespread subclades in the phylogenetic tree as observed in mango [13] and sugarcane [29]. Clade 3 is mostly made up of RLPs with subclades from other RGA domains, particularly TM-CC, which created a large, nested subclade. Clade 4 is made up of NBS-containing proteins (NBS, CNL, TNL, CN, TN, NL, TX, and other NBS proteins), with minor subclades including RLP and RLK.

One of the selective forces that have been ascribed to the diversity and evolutionary pattern of RGAs in plants is the co-evolutionary “arms race” between the host plant and associated pests and diseases to overcome each other [45, 46]. The diversity of RGAs have also been correlated to climatic conditions (e.g., temperature, rainfall, humidity) that promote disease growth and spread [47]. The prevalence of whole-genome duplications and genomic reorganizations in ancient periods has also been linked to the expansion of RGA families and the emergence of novel gene functions in plants [48]. These are some of the factors that may have impacted the evolutionary structure of RGAs of durian, which is mostly cultivated in tropical environments.

## Conclusion

In this study, we successfully identified and characterized the genome-wide RGAs of durian through mining of the currently available reference genome from Musang King cultivar. A considerable number of genome-wide RGAs (2586) were identified in durian which were broadly classified into four major families based on their conserved structural features, i.e., 778 NBS-encoding proteins, 1377 RLKs, 246 RLPs, and 185 TM-CC proteins. The RGAs were functionally annotated to provide a better understanding of their associated MFs, BPs, and CCs, as well as insights into the overall functional response of durian to insect pests and diseases. Furthermore, the investigation of the evolutionary relationships and diversity of RGAs serves as an invaluable reference in the design of framework for genetic improvement of durian. With this, the thorough RGA analysis performed in this work offers a vital resource for genetic, agronomic, and other biological studies on this important tropical fruit crop.

## Supplementary Information

**Additional file 1.**  FASTA protein sequences of Resistance Gene Analogs identified in durian. 

**Additional file 2.**  Direct GO counts of Molecular Function (MF), Biological Process (BP) and Cellular Component (CC) of durian genome-wide RGAs. 

**Additional file 3.** BLAST2GO annotation results of identified RGA proteins.

**Additional file 4.** Bayesian Information Criterion (BIC) scores for the selection of best substitution model for phylogenetic tree construction. 

## Data Availability

The datasets supporting the conclusions of this article are included within the article (and its additional files).

## Abbreviations

RGA: Resistance gene analog
PRRs: Pathogen recognition receptors
R: Resistance
NBS: Nucleotide binding site proteins
NL: NBS-leucine rich repeat proteins
CNL: Coiled-coil NBS-LRR
TNL: Toll/interleukin-1 receptor NBS-LRR
CN: Coiled-coil NBS
TN: Toll/interleukin-1 receptor NBS
TX: Toll/interleukin-1 receptor with unknown domain
RLP: Receptor-like proteins
RLK: Receptor-like kinases
TM: Transmembrane
CC: Coiled coil
LRR: Leucine-rich repeat
TIR: Toll/interleukin-1 receptor
LysM: Lysin motif
NGS: Next-generation sequencing
GO: Gene ontology
MF: Molecular function
BP: Biological processes
CC: Cellular component
